# Psychotherapeutic Interventions in Humanitarian Settings

**DOI:** 10.1192/j.eurpsy.2025.117

**Published:** 2025-08-26

**Authors:** S. Tanyeri Kayahan

**Affiliations:** Psychiatry, Republic of Türkiye Ministry of Health Yalvaç State Hospital, Isparta, Türkiye

## Abstract

**Abstract:**

Humanitarian crises result from various events threatening many individuals’ health, safety, and well-being. The frequency of public health crises has increased within the last decades, posing significant risks to health, particularly for people in low- and middle-income countries. Social determinants such as discrimination, poverty, and violence are risk factors for mental health conditions. People living through humanitarian crises might experience mental distress, with challenging circumstances that lead to vulnerability to developing mental disorders, including depression, anxiety disorders, and post-traumatic stress disorder.

Psychotherapy in humanitarian settings mainly refers to providing psychological support and treatment to individuals affected by wars and armed conflicts, famine, and natural disasters such as earthquakes, hurricanes, or floods. Humanitarian settings pose unique challenges to the delivery of mental health services due to disrupted infrastructure, cultural differences, and the complex needs of affected individuals. Therefore, psychotherapeutic interventions in humanitarian settings should recognize the psychological and social aspects of mental well-being for prevention and treatment. The psychological components of such interventions are related to the mental and emotional state of the person. Social components include efforts to strengthen social support and interpersonal skills, promoting positive aspects of mental health.

In humanitarian settings, mental health care is often integrated with other services, such as education and social services. This holistic approach ensures that mental health is addressed with basic needs like food, shelter, and safety. Another critical aspect is ensuring the safety and mental well-being of therapists at risk in conflict zones. Although limited evidence shows their effects on better mental health outcomes, several therapeutic modalities are frequently used in humanitarian settings, adapted to unique circumstances. Those include cognitive-behavioral therapy, group therapy, trauma-focused psychotherapy, mindfulness and stress reduction, narrative therapy, and interpersonal psychotherapy.

In conclusion, psychotherapy in humanitarian settings plays a crucial role in addressing the mental health needs of individuals affected by crises. It requires flexibility, cultural sensitivity, and a multidisciplinary approach to overcome the challenges posed by these environments. Digital platforms and web-based services might offer accessible and resourceful therapy options. However, further research is needed to evaluate the effectiveness of psychosocial interventions in such settings. Efforts to expand real-world solutions in mental health services, build local capacities, and integrate psychosocial support into broader humanitarian assistance in low-resourced settings are essential to fostering recovery and resilience in affected populations.

**Disclosure of Interest:**

None Declared

